# Impact of construal level manipulations on delay discounting

**DOI:** 10.1371/journal.pone.0177240

**Published:** 2017-05-23

**Authors:** Richard Yi, Allison Stuppy-Sullivan, Alison Pickover, Reid D. Landes

**Affiliations:** 1 Department of Health Education and Behavior, University of Florida, Gainesville, FL, United States of America; 2 Department of Psychology, Yale University, New Haven, CT, United States of America; 3 Department of Psychology, University of Memphis, Memphis, TN, United States of America; 4 Department of Biostatistics, University of Arkansas for Medical Sciences, Little Rock, AR, United States of America; Johns Hopkins School of Medicine, UNITED STATES

## Abstract

Construal Level Theory states that psychologically proximal outcomes are construed concretely while psychologically distal outcomes are construed abstractly. Previous research suggests that the principles of Construal Level Theory can be applied to enhance self-control, as measured by delay discounting. The present studies replicate and expand on this work by examining whether theory-informed priming manipulations lead to delay discounting reductions in a repeated-measures design. Study 1 conceptually replicated previous work, with reduced delay discounting observed as a function of thinking abstractly. Studies 2 and 3 expanded on this work by reinterpreting (a) preference for immediate outcomes as preference for outcomes that are construed concretely, and (b) dispreference for delayed outcomes as dispreference for outcomes that are construed abstractly. Study 2 provided support for the first interpretation, as reduced delay discounting was observed as a function of thinking concretely about the future. Study 3 provided support for the second interpretation, as reduced delay discounting was observed as a function of thinking abstractly about the present. In studies 1 and 3, significant condition × order interactions were observed. In all three studies, the same impact of order of exposure to priming manipulation was observed, indicating specific carryover effects.

## Introduction

### Delay discounting

In the classic delay of gratification studies [1], a child has the choice between one marshmallow immediately and two marshmallows after waiting a period of time. Many of life’s choices involve similar decisions between smaller-immediate and larger-delayed rewards, and an important process in this choice scenario is the relative valuation of immediate and delayed outcomes: in other words, delay discounting. Assessed using choice procedures where individuals indicate preference between smaller-immediate and larger-delayed rewards, delay discounting refers to the reduction in the value of future outcomes as a function of delay; steep delay discounting indicates that rewards quickly lose value with increasing delay (diminished self-control), while shallow delay discounting indicates that rewards largely maintain value (enhanced self-control).

Delay discounting has received significant attention as a measure of self-control; steep (i.e., high) delay discounting is associated with many problematic behaviors, including risky sexual behavior, criminal offending, and addiction disorders [2–4]. Importantly, shallow (i.e., low) delay discounting is associated with lower rates of smoking initiation and alcohol consumption [5–6], and positive drug treatment outcomes [7–9]. It has been hypothesized that an excessively high delay discounting is a trans-disease process that behaviorally contributes to the development of countless diseases [10]. Accordingly, methods that reduce delay discounting could have significant impact on the well-being of individuals and societies. Given the real-world consequences associated with this trans-disease processes, there is a growing interest in exploring approaches that reduce delay discounting; the influential *Construal Level Theory* [11–12] may provide an effective approach to this end.

### Construal Level Theory

Relevant for delay discounting, Construal Level Theory states that (a) various dimensions of distance can be reduced to a single dimension of psychological distance, and (b) representations of proximal events are characterized by concrete, low-level construal while mental representations of psychologically distal events are characterized by abstract, high-level construal. In other words, level of construal covaries with psychological (e.g., temporal) distance. Importantly, the relationship between psychological distance and level of construal is bidirectional, such that perception of distance impacts level of construal and vice versa [13].

Previous research has explored the implications of Construal Level Theory on measures associated with self-control, including task persistence on a handgrip task, evaluations of temptations that undermine studying, and preference for healthy snacks [14–15]. This line of research largely indicates that abstract construal promotes self-control [12, 15–16], suggesting that priming of abstract thinking activates perception of psychological distance, which promotes self-controlled behavior.

One study by Fujita and colleagues [14] explicitly evaluated the impact of construal level on subjective value of delayed outcomes. Following exposure to a construal manipulation similar to that of Vallacher and Wegner [17–18] in which participants answered questions about *why* (abstract construal) or *how* (concrete construal) they maintained good physical health, participants indicated the monetary value of four hypothetical purchases to occur right now or in the future. By calculating difference scores between values of the same immediate and delayed purchases, proxy measures of the relative preference for the immediate and delayed commodities were determined. Consistent with the established literature on Construal Level Theory and self-control, a diminished preference for immediate commodities was observed following abstract construal, compared to following concrete construal.

Given the limited application to date of Construal Level Theory to delay discounting, further exploration offers substantial promise an as approach to reduce delay discounting and associated health-compromising behaviors. Accordingly, the purpose of the present series of studies was to conceptually replicate and extend the previous work applying Construal Level Theory to delay discounting. The first study examined the impact of a previously-validated construal manipulation [19] on delay discounting of money. The second and third studies explored a modified interpretation of Construal Level Theory as applied to delay discounting; that relative preference for immediate outcomes and dispreference for delayed outcomes (high delay discounting) can be interpreted as relative preference for concrete outcomes and/or relative dispreference for abstract outcomes. Accordingly, concrete construal of delayed outcomes should increase preference for delayed outcomes (study 2) and abstract construal of immediate outcomes should decrease preference for immediate outcomes (study 3), both resulting in reduced delay discounting.

### Study 1

Much of the research applying Construal Level Theory to self-control indicates that self-control is enhanced due to abstract construal, and the present study applies this work to the delay discounting of money. Within a repeated-measures counterbalanced design, participants engaged in concrete vs abstract construal while completing a delay discounting task. Level-of-construal was manipulated using a task similar to the mindset induction procedure of Freitas and colleagues [19].

## Method

### Participants

Fifty undergraduate psychology students from the University of Maryland enrolled in the study, provided written informed consent, and received course credit for participation. Recruitment occurred between 2/12/2014 and 7/8/2014, and the recruitment target number was informed by previous research that experimentally applied construal manipulations in the examination of delay discounting and social discounting [20–21]. Approximately half of the participants were randomized to the abstract construal condition first, with the remaining participants randomized to the concrete construal condition first. Four participants were removed from analysis (two participants did not follow instructions, and two received incorrect procedures due to experimenter error); forty-six remaining participants provided complete datasets.

### Materials

#### Abstract construal condition

Participants completed four blocks of a paper questionnaire in which they were presented with a focal action (call a high school friend, buy a computer, do laundry, subscribe to a newspaper) and asked *why* they would engage in that activity. After providing a response to each initial focal action, participants were then asked to state *why* they would engage in each previous response three more times. Thus, participants provided four answers to “why” questions in this way for each block.

#### Concrete construal condition

Participants completed four blocks of a paper questionnaire in which they were presented with a focal action (the same as in the abstract construal condition: call a high school friend, buy a computer, do laundry, subscribe to a newspaper) and asked *how* they would engage in that activity. After providing a response to each initial focal action, participants were then asked to state *how* they would engage in each previous response three more times. Thus, participants provided four answers to “how” questions in this way for each block.

#### Delay discounting task

A computerized binary-choice delay discounting task [22–23] was administered for hypothetical money. On the first trial, two outcomes were presented on the screen: $50 available immediately and $100 available after a specified delay. On each subsequent trial across a six-trial sequence, the outcome available immediately was adjusted based on the participant’s choice on the previous trial: it was reduced by 50% of the previous adjustment if the immediate outcome was selected, and increased by 50% of the previous adjustment if the delayed outcome was selected. This six-trial sequence was completed at each of four delays (1 week, 6 months, 1 year, and 5 years) to determine a present, subjective value (i.e., indifference point) of $100 at each delay. Delays were always presented in increasing order.

### Procedure

The study protocols for all studies reported here were approved by the University of Maryland Institutional Review Board. Participants completed two 30-minute sessions, separated by one week. Order was counterbalanced, such that approximately half of the participants were randomly assigned to receive the sessions in the Abstract-Concrete order and half in the Concrete-Abstract order. In each session, the four blocks of construal manipulations were interweaved with the four delays of the delay discounting task such that each block of how/why questions preceded one series of delay discounting binary choice questions at each delay. For example, in the Abstract condition, a block of four *why* questions regarding calling a high school friend was followed by assessment of delay discounting for $100 delayed by 1 week; in the Concrete condition, a block of four *how* questions regarding calling a high school friend was followed by assessment of delay discounting for $100 delayed by 1 week. During each session, participants completed either the Abstract or Concrete construal manipulation, along with the computerized delay discounting task. The pairing of each block of construal questions and the delay in the delay discounting task was fixed such that participants were exposed to the same sequence of construal question blocks between-conditions and between-subjects. All measures and manipulations are fully reported here.

## Analytic plan

### Manipulation check

To ensure that participants were able to engage in concrete versus abstract thinking, two blind raters coded responses to the construal manipulation, as outlined previously [14]. For each block, subsequent construal responses were rated *+1* for responses that were superordinate to or more abstract than the previous response (e.g., *to wear clean clothes* in response to *why* do laundry), *-1* for responses that were subordinate to or more concrete than the previous response (e.g., *separate dirty clothes into piles by color* in response to *how* to do laundry), and *0* if the response was neither superordinate or subordinate to the previous response. The rationale for this scoring system is based on the assumption that a response that is more abstract than the previous response contributes to the overall level of abstractness, one that is less abstract detracts from overall abstractness, and one that is neither more nor less abstract does not affect overall abstractness. The scores associated with the four responses within a block were then summed, resulting in a score ranging from -4 to +4. These scores were averaged across the four blocks, with positive/negative scores indicating net abstract/concrete construal, respectively. Given acceptable inter-rater reliability (*ICC* = 0.71 and 0.87 for Abstract and Concrete conditions, respectively), construal scores from the two raters were averaged within each subject × condition combination.

### Delay discounting

Nonlinear regression was used to obtain estimated delay discounting parameters for each participant under each condition, based on the hyperbolic function [24]:

$$V_{d}=\frac{V}{1+kD}$$

where *V*_*d*_ is the discounted value of an outcome, *V* is the undiscounted value, *D* is delay in days, and *k* provides a measure of the degree to which the value of a reward is discounted when it is delayed (i.e., discount rate). Low values of *k* indicate low delay discounting (slow loss of value resulting from delay, i.e., enhanced self-control). The distributions of delay discounting values (*k*) were positively skewed, so natural logarithm transformations were conducted in order to normalize the distributions. These transformed measures of delay discounting (ln(*k*)) were analyzed within a repeated-measures analysis of variance (ANOVA), with construal condition (Abstract vs. Concrete) as a within-individual factor, order (Abstract-Concrete vs. Concrete-Abstract) as an among-individual factor, and the interaction of condition and order. An exchangeable (a.k.a. compound symmetric) covariance structure was used to model the within-individual covariance. These analyses were conducted using SPSS v. 21 as repeated-measures general linear model, and effect sizes (η^2^_G_) were computed as in Olejnik and Algina [25]. To examine the association of the construal scores from the manipulation check with measures of delay discounting, Spearman correlations were conducted within each construal condition.

## Results

Table 1 summarizes participant demographics (age, sex, race, year in school, SAT score, and GPA). These variables were not found to be associated with delay discounting, and therefore were not included in the main analysis. All but two participants completed the sessions exactly one week apart.

**Table 1:** Participant characteristics.

	*n (%)*		
	Study 1 *N = 46*	Study 2 *N* = 42	Study 3 *N* = 47
Gender			
Male	10 (21.7)	13 (31.0)	8 (17.0)
Female	36 (78.3)	29 (69.0)	39 (83.0)
Age			
*M (SD)*	19.2 (1.9)	20.4 (1.7)	19.6 (2.5)
(Min, Max)	(16, 28)	(18, 26)	(16, 31)
Race			
White	22 (47.8)	19 (45.2)	17 (36.2)
Black/African-American	10 (21.7)	5 (11.9)	13 (27.7)
Hispanic/Latino	3 (6.5)	5 (11.9)	4 (8.5)
Asian/Southeast Asian	9 (19.6)	12 (28.6)	10 (21.3)
Native American/American Indian	0 (0.0)	0 (0.0)	1 (2.1)
Other	2 (4.3)	1 (2.4)	2 (4.3)
Academic Rank			
Freshman	18 (39.1)	9 (21.4)	15 (31.9)
Sophomore	7 (15.2)	12 (28.6)	14 (29.8)
Junior	17 (37.0)	13 (31.0)	9 (19.1)
Senior	4 (8.7)	8 (19.0)	8 (17.0)
Unreported	0 (0.0)	0 (0.0)	1 (2.1)
SAT Math			
*M (SD)*	633.6 (91.4)	590.5 (115.0)	650.4 (75.4)
(Min, Max)	(420, 800)	(300, 800)	(510, 780)
SAT Verbal			
*M (SD)*	649.6 (100.0)	647.0 (95.0)	654.4 (689.6)
(Min, Max)	(350, 800)	(360, 800)	(520, 790)
GPA			
*M (SD)*	3.40 (0.46)	3.23 (0.47)	3.40 (0.52)
(Min, Max)	(2.2, 4.2)	(2.0, 3.93)	(2.0, 4.8)

### Manipulation check

Construal scores were compared between the Abstract and Concrete construal conditions. As expected, *why* questions generated responses that reflected abstract construal with a mean of +2.51, and *how* questions reflected concrete construal with a mean of –3.40 (paired-*t*(45) *=* 21.9, *p* < .001, see Table 2).

**Table 2:** Construal ratings for each condition in each study. Negative values indicate concrete construal and positive values abstract construal.

Study	Conditions	N	Mean	sd
1	Concrete	46	-3.40	1.18
	Abstract		+2.51	1.48
2	Concrete/Present	42	-1.78	0.33
	Concrete/Future		-1.50	0.44
3	Abstract/Future	47	+3.90	0.35
	Abstract/Present		+3.94	0.21

### Delay discounting

**Fig 1:**
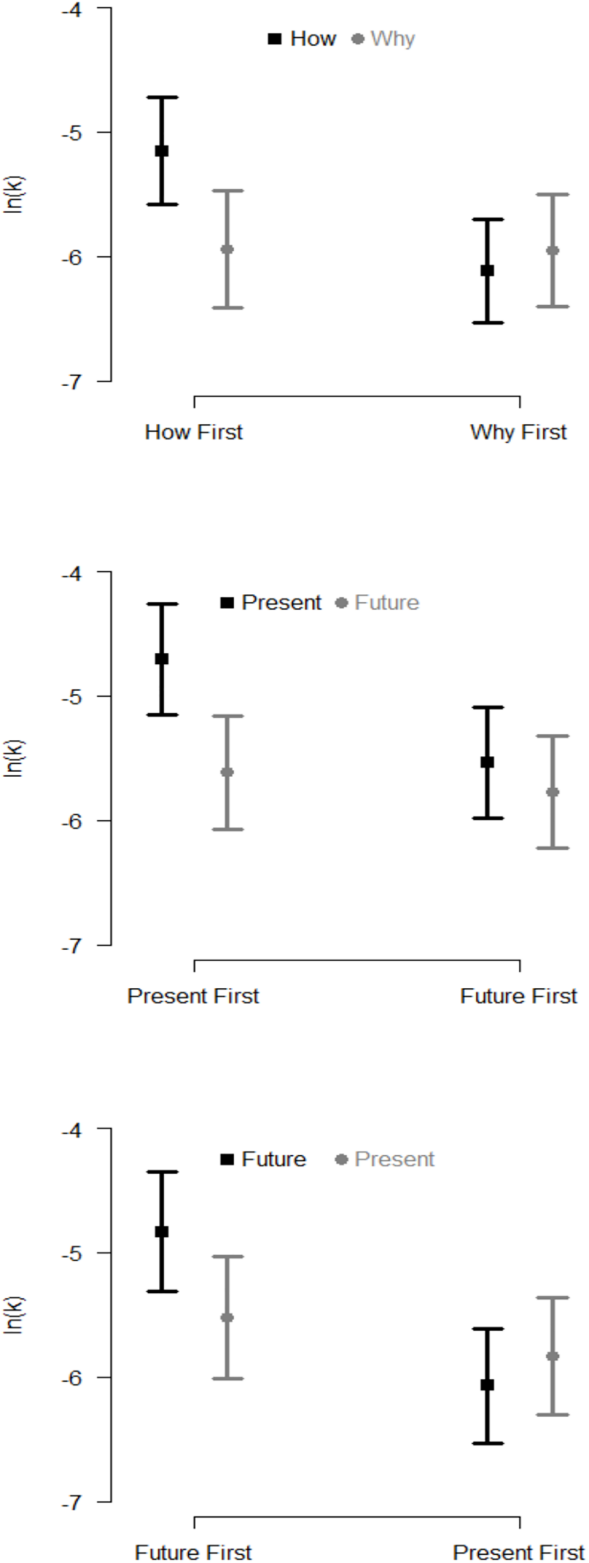
Mean (±SE) log-transformed delay discounting (ln-*k*) in the two conditions of each study, by order of exposure to conditions. Study 1 (top panel): *Why* (gray bars) and *How* (black bars) conditions. The condition × order interaction is statistically significant, with lower delay discounting in the *Why* condition compared to *How* condition when the *How* condition occurred first. Study 2 (middle panel): *Concrete/Future* (gray bars) and *Concrete/Present* (black bars) conditions. Lower delay discounting was observed in the *Concrete/Future* condition, with no interaction. However, as with study 1, delay discounting was lower in the *Concrete/Future* condition compared to *Concrete/Present* when the *Concrete/Present* condition occurred first. Study 3 (bottom panel): *Abstract/Present* (gray bars) and *Abstract/Future* (black bars) conditions. As with study 1, the condition × order is statistically significant. As with studies 1 and 2, delay discounting was lower in the *Abstract/Present* condition compared to *Abstract/Future* when the *Abstract/Future* condition occurred first.

Goodness-of-fit of the hyperbolic discounting model to the data was assessed with R^2^ and root mean squared error (RMSE). R^2^ is a biased goodness-of-fit measure frequently reported in delay discounting reports, and though not appropriate for nonlinear regressions [26], it is reported here for consistency with the established literature (${\bar{X}}_{R}{}^{2}$ = .788, *SD*_*R*_^*2*^ = .300). RMSE was ${\bar{X}}_{\mathrm{MSE}}$ = .075, *SD*_*MSE*_ = .053. Delay discounting between conditions were positively correlated (Pearson’s *r*(44) = +0.75; 95% CI: (0.59, 0.86)).A 2×2 ANOVA examined the effects of condition, order, and their interaction, and revealed a significant interaction (*F*(1,44) = 5.18, *p* = .028), with no significant main effects (both *p*’s > 0.14; Fig 1, top panel). Simple effects tests revealed significantly lower delay discounting in the Abstract condition compared to Concrete condition (mean difference = .79, 95% CI: (0.18, 1.41), *t*(44) = -2.60, *p* = .013; η^2^_G_ = .133) only when the Concrete condition occurred first; no effect on delay discounting was observed when the Abstract condition occurred first (mean difference = 0.17, 95% CI: (-0.75, 0.42), *t*(44) = 0.57 *p* = .571; η^2^_G_ = .007). Correlations conducted between construal scores and delay discounting revealed no significant associations in the Abstract (Spearman’s *r*(44) = +.25, *p* = .10) nor Concrete (Spearman’s *r*(44) = -.10, *p* = .51) construal conditions.

## Discussion

Consistent with the developing research applying Construal Level Theory to self-control [12, 15–16], the present study indicates that activation of abstract construal resulted in delay discounting reductions. Most directly, this work is a conceptual replication of study 1 of Fujita and colleagues [14] using different operationalizations of both independent and dependent variables. In that study, level-of-construal was manipulated using a means/ends task and delay discounting was measured as the difference in immediate/delayed purchase value of various commodities. The present study manipulated level of construal using a variation of the means/ends task and measured delay discounting using a well-established procedure for hypothetical money outcomes.

The alignment between the construal conditions and the corresponding construal scores suggests that the manipulation was effective in priming the appropriate level of construal. Combined with the observed difference in delay discounting as a function of construal condition, this would suggest that delay discounting could be a function of the degree abstractness or concreteness. However, the present results were not able to directly establish this relation; no significant relation was observed between construal score and delay discounting in the bivariate correlations. Given that the range of construal scores were fairly restricted within-condition, this result is not surprising.

The present results are qualified by the impact of order of exposure to the construal conditions. Specifically, the predicted impact of construal on delay discounting was only observed when concrete construal preceded abstract construal, but not in the reverse order. In order to explore the possibility that the observed construal × order interaction was spurious, as well as to expand the application of Construal Level Theory to enhance self-control, two additional studies were conducted. These studies sought to examine the possible effect of *time-construal asynchrony*, in which there is a mismatch between level of construal (concrete versus abstract) and temporal distance (present versus future, respectively).

## Study 2

Despite the abundance of evidence that self-control is enhanced with abstract construal, concrete construal can result in enhanced self-control in some contexts [27]. Specifically applied to delay discounting, a complementary account of Construal Level Theory is that preference for immediate outcomes is associated with concrete construal, and preference for delayed outcomes is associated with abstract construal. Accordingly, activating *concrete* construal for *delayed* outcomes should increase preference for those outcomes, resulting in reduced delay discounting. A developing body of research examining *episodic future thinking*, comparable to concrete construal of future outcomes, is providing increasing support for this idea [20, 28–33]; data indicate that concrete construal of future outcomes results in delay discounting reductions. In order to expand this work, the present study examined the impact of concrete construal of the future on delay discounting using a repeated-measures design that maintains methodological coherence with study 1. Participants completed delay discounting procedures while answering questions designed to prime concrete construal of the future or concrete construal of the present.

## Method

### Participants

Forty-three undergraduate psychology students from the University of Maryland enrolled in the study, provided written informed consent, and received course credit for participation. Recruitment occurred between 4/6/2011 and 5/9/2011, and the recruitment target was informed by study 1. One participant did not provide usable data and was excluded from the analyses; thus forty-two remaining participants provide complete datasets. There was no overlap with participants in study 1.

### Materials

#### Concrete construal of the future (Concrete/Future) condition

Participants completed four blocks of an open-ended paper questionnaire, designed to prime episodic, concrete thinking about the future. Each block was comprised of five fill-in-the-blank questions regarding specific events/activities (having lunch, visiting a website, engaging in a leisurely activity, and having a conversation) that would occur 1 week, 6 months, 1 year, and 5 years from now (see S1 Appendix for all items). The time qualifiers associated with each block of episodic questions coincided with the delays in the delay discounting task.

#### Concrete construal of the present (Concrete/Present) condition

Participants completed four blocks of an open-ended paper questionnaire, designed to prime episodic, concrete thinking about the present. Each block was comprised of five fill-in-the-blank questions regarding specific events/activities, and the blocks of questions were identical to those used in the Concrete/Future condition with the exception of the time qualifier; the time-frame was “today” for all questions.

#### Delay discounting task

The computerized delay discounting task was identical to the one administered in Study 1. In the Concrete/Future condition, the block of episodic questions regarding events 1 week, 6 months, 1 year, and 5 years in the future preceded assessment of delay discounting for delayed rewards at 1 week, 6 months, 1 year, and 5 years, respectively. In the Concrete/Present condition, assessment of delay discounting for all delays was preceded by blocks of episodic questions regarding today.

### Procedure

Participants completed two 30-minute sessions, separated by one week. In each session, the four blocks of construal manipulation questions were interweaved with the four delays of the delay discounting task. In the Concrete/Future condition, questions regarding events 1 week, 6 months, 1 year, and 5 years in the future preceded assessment of delay discounting at delays 1 week, 6 months, 1 year, and 5 years, respectively. In the Concrete/Present condition, questions regarding events today preceded assessment of delay discounting at all delays. Approximately half of the participants were exposed to the Concrete/Future condition in the first session, followed by the Concrete/Present condition in the second session. The remaining participants experienced the reversed order of construal conditions. The pairing of each block of construal questions and the subsequent delay in the delay discounting task was fixed such that participants were exposed to the same sequence of construal question blocks between-conditions and between-subjects. All measures and manipulations are fully reported here.

## Analytic plan

### Manipulation check

To ensure that participants were able to engage in concrete thinking of future and present events, two blind raters coded responses to the construal manipulation. Responses were coded on a 7-point scale ranging from -3 (completely concrete; e.g., *large green salad with vinaigrette dressing* in response to *what will you have for lunch*?) to +3 (completely abstract; e.g., *food* in response to *what will you have for lunch*?). For each rater, the 20 scores (4 blocks × 5 questions) were averaged within each subject × condition combination. Given acceptable inter-rater reliability (*ICC* = 0.77 and 0.63 for Concrete/Future and Concrete/Present conditions, respectively), construal scores from the two raters were averaged within each subject × condition combination.

### Delay discounting

As in Study 1, delay discounting measures were calculated, transformed, and analyzed in a repeated-measures ANOVA. The ANOVA had construal condition (Concrete/Future vs. Concrete/Present) as the within-individual factor, order (Concrete/Future-Concrete/Present vs. Concrete/Present-Concrete/Future) as an among-individual factor, and the interaction of condition and order. To examine the association of the construal scores with delay discounting, Spearman correlations were conducted within each construal condition.

## Results

Participant characteristics are summarized in Table 1. Demographic variables were not found to be associated with delay discounting, and therefore were not included in the main analysis. All but six participants completed the sessions exactly one week apart.

### Manipulation check

Construal scores were compared between the Concrete/Future and Concrete/Present conditions. Participants provided concrete responses in both conditions, with negative mean ratings in both Concrete/Future and Concrete/Present conditions (-1.50 and -1.78, respectively), though scores were significantly more concrete in the Concrete/Present condition (paired-*t*(41) *=* -3.64, *p* = .001; see Table 2).

### Delay discounting

Goodness-of-fit measures were appropriate (${\bar{X}}_{R}{}^{2}$ = .782, *SD*_*R*_^*2*^ = .306; ${\bar{X}}_{\mathrm{RMSE}}$ = .080, *SD*_*RMSE*_ = .051). The 2 × 2 ANOVA examining the effects of condition, order, and their interaction revealed that delay discounting was lower in the Concrete/Future condition compared to the Concrete/Present condition (-5.69 vs. -5.12, 95% CI: (0.01, 1.14); *F*(1,40) = 4.16, *p* = .048, η^2^_G_ = .094). Neither the main effect of order nor interaction were significant (both *p* > .237, see Fig 1, middle panel).

Despite the fact that no significant interaction was observed, based on visual inspection of the cell means as well as the condition × order interaction observed in study 1, simple effects analyses were conducted comparing conditions separately for each order. Results were consistent with study 1: an effect of condition was observed in the Concrete/Present-then-Concrete/Future order (*t*(40) = 2.29, *p* = .027, η^2^_G_ = .116, difference = 0.91, 95% CI: (0.11, 1.71)), but was not observed in the Concrete/Future-then-Concrete/Present order (*t*(40) = 0.59, *p* = .556, η^2^_G_ = .009, difference = 0.24, 95% CI: (-0.57, 1.04)).

Correlations conducted between construal scores and delay discounting revealed a non-significantly negative association in the Concrete/Present condition (Spearman’s *r*(40) = -.28, *p* = .07). However, a significantly negative association in the Concrete/Future condition (Spearman’s *r*(40) = -.47, *p* = .002) indicated that more concrete answers about the future was associated with greater delay discounting.

## Discussion

Broadly, the results of study 2 are consistent with the assertion that tenets of Construal Level Theory can be applied to reduce delay discounting. Similar to observations from previous research [28–33], concrete construal of the future (i.e., episodic future thinking) increased relative preference for future outcomes and reduced delay discounting, compared to concrete construal of the present. In contrast to study 1, there was no significant condition × order interaction. However, our planned analysis of the effect of condition separately by order indicated a similar pattern of results to study 1. Specifically, the decreasing effect on delay discounting by the construal manipulation was observed going from the Concrete/Present condition to the Concrete/Future condition, but not in the reverse order.

Construal scores indicated that participants were able to engage in concrete thinking in both conditions, though scores were more concrete when thinking about the present than the future. This was not surprising, as thinking concretely about the present is consistent with how humans typically function according to Construal Level Theory, whereas thinking concretely about the future is less common and may represent a challenge. The examination of the association between construal scores and delay discounting within each condition revealed mixed results. The correlation between these variables in the Concrete/Present condition revealed a negative relation that fell just short (*p* = .07) of reaching the conventional threshold for statistical significance. A negative correlation was expected in this condition, indicating that the degree of thinking concretely about the present (more concrete = more negative) increases preference for present outcomes (hence increase delay discounting). However, the statistically significant negative correlation in the Concrete/Future, indicating that thinking concretely about the future decreased preference for future outcomes, is contrary to what was expected. As the meaningfulness of these contradictory associations is unclear in isolation, we reserve further discussion of this finding to the General Discussion.

A challenge to the interpretation of the between-condition effect of the construal manipulation on delay discounting, however, is the possibility that simply thinking (concretely or abstractly) about the future increased preference for delayed outcomes. In other words, any level-of-construal thinking of the future highlights delayed outcomes, and reduction in delay discounting is simply due to increased attention to the future. In order to address this possibility, as well as to build on the application of Construal Level Theory to delay discounting, a third study was conducted to examine the impact of abstract construal of the present.

## Study 3

A corollary to the interpretation of Construal Level Theory that elevated delay discounting indicates relative preference for concreteness is that it also indicates relative dispreference for abstractness. According to this interpretation, abstract construal of the present should result in relative dispreference for immediate outcomes and reduce delay discounting; this is consistent with time-construal asynchrony. Results consistent with this interpretation of Construal Level Theory would not only provide support for another time-construal asynchrony approach to reduce delay discounting, but also address the concern in study 2 that simply thinking about the future/present, rather than the level-of-construal, impacts preference for future/present outcomes (i.e., impacts delay discounting).

## Method

### Participants

Fifty undergraduate psychology students from the University of Maryland enrolled in the study, provided written informed consent, and received course credit for participation. Recruitment occurred between 10/2/2014 and 1/23/2014, and the recruitment target was informed by studies 1 and 2. Incomplete data from three participants were excluded from the analyses (one participant did not complete the study, two participants completed construal questions and discounting items in the wrong order). Forty-seven remaining participants provided complete datasets. There was no overlap with participants in studies 1 nor 2.

### Materials

#### Abstract construal of the present condition (Abstract/Present)

Participants completed four blocks of a paper questionnaire in which they were presented with a version of the *abstract* version of the mindset induction task [19] with present time qualifiers; participants were presented with a focal action and asked why they would/did engage in that activity *today*. After providing a response to the initial action, participants were then asked to state *why* they would engage in the previous response three more times, each time with the time qualifier of *today*. Participants provided four answers to “why” questions in this way for each block.

#### Abstract construal of the future condition (Abstract/Future)

Participants completed four blocks of a paper questionnaire in which they were presented with the same *abstract* version of the mindset induction task, with future time qualifiers (1 week, 6 months, 1 year, and 5 years). Participants were presented with a focal action and asked why they would/did engage in that activity at a point in the future. After providing a response to the initial action, participants were then asked to state *why* they would engage in their previous response three more times, each time with the future time qualifier. Participants provided four answers to “why” questions in this way for each block.

#### Delay discounting task

The computerized delay discounting task was identical to the one administered in studies 1 and 2. In the Abstract/Present condition, discounting questions for all delays were preceded by mindset induction questions regarding today. In the Abstract/Future condition, mindset induction questions regarding events 1 week, 6 months, 1 year, and 5 years in the future preceded assessment of delay discounting for delayed rewards at 1 week, 6 months, 1 year, and 5 years, respectively.

### Procedure

Participants completed two 30-minute sessions, separated by one week. In each session, the four blocks of construal manipulation questions were interweaved with the four delays of the delay discounting task. In the Abstract/Present condition, questions regarding events today preceded assessment of delay discounting at all delays. In the Abstract/Future condition, questions regarding events 1 week, 6 months, 1 year, and 5 years in the future preceded assessment of delay discounting at 1-week, 6-month, 1-year, and 5-year delays, respectively. Approximately half of the participants were exposed to the Abstract/Present condition in the first session, followed by the Abstract/Future condition in the second session. The remaining participants experienced the reversed order of construal conditions. The pairing of each block of construal questions and the subsequent delay in the delay discounting task was fixed such that participants were exposed to the same sequence of construal question blocks between-conditions and between-subjects. All measures and manipulations are fully reported here.

## Analytic plan

### Manipulation check

To ensure that participants were able to engage in abstract thinking of present and future actions, two blind raters coded responses to the construal manipulation. As in Study 1, two blind raters scored the mindset induction task using the method previously proposed [14]. Abstractness ratings for each focal action were averaged for each condition, and higher scores indicated greater levels of abstractness. Given acceptable inter-rater reliability (ICC = 0.97 and 0.91 for Abstract/Present and Abstract/Future conditions, respectively), construal scores from the two raters were averaged within each subject × condition combination.

### Delay discounting

As in Studies 1 and 2, delay discounting measures were calculated, transformed, and analyzed using repeated-measures ANOVA. The ANOVA had construal condition (Abstract/Present vs. Abstract/Future) as the within-individual factor, order (Abstract/Present-Abstract/Future vs. Abstract/Future-Abstract/Present) as an among-individual factor, and the interaction of condition and order. To examine the association of the construal scores with delay discounting, Spearman correlations were conducted within each construal condition.

## Results

Participant characteristics are summarized in Table 1. Demographic variables were not found to be associated with delay discounting, and therefore were not included in the main analysis. All but two participants completed the sessions exactly one week apart.

### Manipulation check

Comparing mean construal between the two conditions, scores indicate abstract construal, with high mean abstractness ratings in both Abstract/Present and Abstract/Future conditions (+3.94 and +3.90, respectively), and no significant difference between them (paired-*t*(46) *=* 0.82, *p* = .415, see Table 2).

### Delay discounting

Goodness-of-fit measures were (${\bar{X}}_{R}{}^{2}$ = .778, *SD*_*R*_^*2*^ = .336; ${\bar{X}}_{\mathrm{RMSE}}$ = .081, *SD*_*RMSE*_ = .060). The 2 × 2 ANOVA revealed a significant order × condition interaction (*F*(1,45) = 6.97, *p* = .011, η^2^_G_ = .134), with no significant main effects (both *p’s* > 0.215; Fig 1, bottom panel). Simple effects tests revealed significantly lower delay discounting in the Abstract/Present condition compared to the Abstract/Future condition (*t*(45) = 2.73; *p* = .009; η^2^_G_ = .142; difference = 0.69; 95% CI: (0.18, 1.19)), only when the Abstract/Future condition occurred first; no effect of condition on delay discounting was observed when the Abstract/Present condition occurred first (*t*(45) = 1.26; *p* = .329; η^2^_G_ = .021; difference = 0.24; 95% CI: (-0.25, 0.74)).

Noting very little variability in construal scores, correlations conducted between construal scores and delay discounting revealed no significant associations in the Abstract/Present (Spearman’s *r*(45) = +.21, *p* = .16) nor Abstract/Future (Spearman’s *r*(45) = -.80, *p* = .59) conditions.

## Discussion

The results of study 3 remain consistent with the assertion that time-construal asynchrony can be applied to reduce delay discounting. More specifically, the pattern of results is congruent with what was observed in Studies 1 and 2, where order of exposure to Abstract/Present and Abstract/Future conditions impacted the emergence of construal’s effect on delay discounting. The predicted impact of level-of-construal was observed when abstract construal of the future preceded abstract construal of the present, but not in the reverse order. The fact that a similar effect of order was also observed in studies 1 and 2 enhances our confidence that this finding, of a construal effect in one order of exposure but not in the other, is a genuine phenomenon.

Importantly, study 3 addressed a possible interpretational challenge to the results of study 2. If simply thinking about the future increased preference for future outcomes and reduced delay discounting, a lower rate of delay discounting would have been observed in the Abstract/Future condition rather than the Abstract/Present condition. The opposite was observed, and rate of delay discounting was lower in the Abstract/Present condition, thus ruling out the possibility that simple attention to future outcomes increases preference for those outcomes.

Construal scores indicated that participants were able to engage in abstract thinking in both conditions. Similar to study 1, exploration of a continuum between construal scores and delay discounting revealed no significant relationship in a bivariate correlation, with the caveat that the construal scores had very limited variability.

## General discussion

The three studies reported here were designed to explore the applicability of Construal Level Theory to self-control. Delay discounting, which provides an index of the degree to which an individual is willing to wait for a delayed outcome, is associated with behavioral outcomes related to self-control [2–5]. Study 1 was a conceptual replication of previous work suggesting that abstract construal promotes preference for future outcomes and decreases delay discounting. While previous work [14] indicated that high-level construal resulted in less delay discounting of specific commodities, study 1 expands upon this finding using a more-comprehensive measure of delay discounting for (hypothetical) money. Consistent with the extant literature, delay discounting was reduced as a result of abstract construal.

Study 2 expanded on the application of Construal Level Theory, based on the view that preference for immediate outcomes can also be interpreted as preference for concrete construal outcomes. Capitalizing on what we are calling time-construal asynchrony, concrete construal of the future resulted in increased preference for future outcomes and reduced delay discounting, compared to concrete construal of the present. This is consistent with recent work on episodic future thinking [28–33]. Study 3 provides additional evidence for the impact of time-construal asynchrony, with results indicating that abstract construal of the present reduced relative preference for immediate outcomes and decreased delay discounting, compared to abstract construal of the future. We are aware of no previous research that has examined this potential interpretation of Construal Level Theory.

The impact of the construal manipulation was apparent in all studies in one order of exposure to the conditions, but not the other. Interestingly, a nearly identical effect of order was also observed by our group [21] using similar construal manipulations and experimental design to examine the construct of social discounting, which is theoretically [34–35] and empirically [36–37] associated with delay discounting.

This pattern of results is consistent with the suggestion by Malkoc and colleagues [38] that concrete construal of the present is the default level-of-construal. Accordingly, we can reconceptualize the Abstract, Concrete/Future, and Abstract/Present conditions from studies 1, 2, and 3, respectively, as experimental conditions, with the alternative in each study as a control (default) condition. Within this framework, the effective reduction in delay discounting was observed in each study in the experimental condition in the control-then-experimental order but not experimental-then-control order.

Said differently, the reduction in delay discounting was observed when the order of construal was *default-then-switch*, but not *switch-then-default*. To wit, the control condition in the first session simply maintains the default construal level, with the experimental condition in the second session requiring a construal shift. In contrast, the experimental condition in the first session requires a construal shift away from default, with the control condition in the second session requiring a construal return to default. To the extent that a shift away from default results in some degree of learning, initial exposure to the experimental condition could have abated the impact of a subsequent control condition, particularly in the research context of the present study. This effect could be related to the phenomenon of *blocking* [39–40], where an unconditioned response does not occur in the presence of a second conditioned stimulus if it does not provide any additional information than the first conditioned stimulus. Initially identified as a phenomenon in classical conditioning, blocking has been proposed by Malkoc and Zauberman [41] as a phenomenon that could impact a construal manipulation.

In proposing this explanation for the order effect observed in the present research, we do not mean to suggest that a *switch* construal manipulation in session 1 blocked the impact of all stimuli that would result in a return to *default* construal or that the individual behaved in a way completely consistent with the switch construal during the one-week wait until session 2. Rather, to the extent that blocking may have played a role, we believe it is possible that the unique environment of the experimental context served as a discriminative stimulus for the construal manipulations of the present studies.

Because the order effect complicates the interpretation of the present studies (though we believe in an interesting and important way), we also conducted contrasts of experimental and control conditions only when they occurred in the first session, in the absence of potential order effects. These between-groups contrasts had substantially diminished statistical power due to having only half the sample in each condition, and we were likely underpowered to detect a significant difference when only considering the first session of the present experimental design. Nonetheless, all mean differences were in the predicted direction: delay discounting in the experimental condition was lower than delay discounting in the control condition by ln-*k* units of 0.80, 1.07, and 0.91 in studies 1, 2, and 3, respectively.

Some limitations of the studies are noteworthy. The most significant limitation in the present series of studies is the absence of a no-construal condition. Our assumption was that the control condition in each study was consistent with the default level of construal and had no effect on delay discounting. However, without a measure of delay discounting in the absence of any construal manipulation, a viable alternative explanation is that the control conditions across the three studies enhanced preference for immediate outcomes (i.e., increased delay discounting). Indeed, the observed differences between conditions could have occurred because the experimental conditions caused decreases in delay discounting, the control conditions caused increases in delay discounting, or both. Despite theoretical arguments that the control condition simply reinforced default levels of construal, the design of the present studies does not allow us to rule out an unintended effect of the control condition. Future research should be conscientious regarding this alternate interpretation and take steps to address it experimentally. A second limitation involves the use of hypothetical money outcomes. Though previous research indicates no difference in delay discounting of real and hypothetical rewards [42], and recent evidence indicates statistical equivalence [43], we cannot definitively rule out different effects using real rewards. Finally, we do not have an explanation for the significantly negative correlation between construal score and delay discounting observed in the Concrete/Future condition of study 2. The construal scores across studies were primarily meant to serve as imprecise manipulation checks, and the design of the experiments reported here did not allow for much variability of these scores. Given that no other significant association was observed between construal score and delay discounting in any of our studies, this one significant correlation may reflect the inflated type-I error rate resulting from multiple tests. However, the possibility of a less-than-straightforward relation between the degree to which an individual can engage in concrete thinking about the future and degree of preference for future rewards should not be dismissed, and future research should examine this question.

Nonetheless, the implications of the proposed construal manipulation are meaningful. The relative overvaluation of immediate outcomes appears to be associated with countless health-compromising behaviors, perhaps indicating a common contributing etiology [10]. Focus on level-of-construal of immediate and delayed outcomes, perhaps capitalizing on the time-construal asynchrony effect observed in studies 2 and 3, could serve as a foundation for approaches to improve intertemporal decision-making. Importantly, time-construal asynchrony may be a particularly effective approach to reduce delay discounting in individuals who exhibit limited self-control. These individuals functionally exhibit preference for outcomes construed concretely and dispreference for outcomes construed abstractly (according to Construal Level Theory). The present interpretation capitalizes on this bias for concrete construal outcomes to promote self-controlled choice. Success in promoting preference for future outcomes may contribute to positive health and financial improvements and reductions in the economic and societal costs of diminished self-control.

## Supporting information

S1 Appendix Concrete construal items from study 2. (DOCX)

S1 Dataset Complete dataset for all studies. (XLSX)
