# Supplementary material for: Impact of construal level manipulations on delay discounting
Source: PLoS One. 2017 May 23;12(5):e0177240. doi: 10.1371/journal.pone.0177240 (PMC5441631; doi:10.1371/journal.pone.0177240)
Supplement: S1 Appendix — (DOCX) [file pone.0177240.s001.docx]

Appendix

Concrete Construal of the Future/Present Questions: Study 2

Block 1

- At what time will you have lunch (one week from today/today)?
- What will you have for lunch (one week from today/today)?
- Where will you have for lunch (one week from today/today)?
- What beverage will you drink at lunch (one week from today/today)?
- Who will you have lunch with (one week from today/today)?

Block 2

- Name a website that you will visit (six months from today/today).
- Why will you visit this website?
- At what time of day will you visit this website?
- What will the primary purpose of this website be (six months from today/today)?
- Where will you be when you visit this website?

Block 3

- Very briefly describe a leisurely activity that you will participate in (one year from today/today)?
- What will you wear for this activity?
- Who else will be involved?
- Where will this activity take place?
- At what time will this activity take place?

Block 4

- With whom will you have a conversation with (five years from today/today)?
- Where will this conversation take place?
- What will the main topic of this conversation be?
- At what time of day will this conversation take place?
- Why will this conversation take place?
